# Visualization of the Nucleolus Using Ethynyl Uridine

**DOI:** 10.3389/fpls.2018.00177

**Published:** 2018-02-16

**Authors:** Martina Dvořáčková, Jiří Fajkus

**Affiliations:** ^1^Mendel Centre for Plant Genomics and Proteomics, Central European Institute of Technology, Masaryk University, Brno, Czechia; ^2^Laboratory of Functional Genomics and Proteomics, National Centre for Biomolecular Research, Faculty of Science, Masaryk University, Brno, Czechia; ^3^Institute of Biophysics, Academy of Sciences of the Czech Republic, Brno, Czechia

**Keywords:** nucleolus, nucleus, transcription, *Arabidopsis thaliana*, click iT

## Abstract

Thanks to recent innovative methodologies, key cellular processes such as replication or transcription can be visualized directly *in situ* in intact tissues. Many studies use so-called click iT chemistry where nascent DNA can be tracked by 5-ethynyl-2′-deoxyuridine (EdU), and nascent RNA by 5-ethynyl uridine (EU). While the labeling of replicating DNA by EdU has already been well established and further exploited in plants, the use of EU to reveal nascent RNA has not been developed to such an extent. In this article, we present a protocol for labeling of nucleolar RNA transcripts using EU and show that EU effectively highlights the nucleolus. The method is advantageous, because the need to prepare transgenic plants expressing fluorescently tagged nucleolar components when the nucleolus has to be visualized can be avoided.

## Introduction

The nucleus, as the most prominent cellular component, represents an important research target, and thus considerable effort has been put into establishing reliable detection methods to track nuclear processes. The most prominent structure in the plant nucleus is the nucleolus, the region where transcription of rRNA genes and processing of their transcripts occur (Stoykova et al., 1985; French and Miller, 1989; Raska et al., 1989; Scheer et al., 1997; Koberna et al., 2002). Due to their unique structure, plant and animal nucleoli have represented an attractive object for microscopy. This can be documented in a number of studies focusing on its structure, metabolism, or DNA and protein components (Jacob and Sirlin, 1964; Bernhard, 1966; Jordan and Luck, 1976; Zankl and Bernhardt, 1977; Ochs et al., 1985; Fakan and Hernandez-Verdun, 1986; Biggiogera et al., 1989; Beven et al., 1995; Kopecny et al., 1996; de Carcer and Medina, 1999).

This article will introduce a method to label nucleolar RNA in the plant model *Arabidopsis thaliana.* The first protocols exploring transcription took advantage of using radioactively labeled [^3^H] uridine detected by autoradiography (Uddin et al., 1984; Wassermann et al., 1988). With the development of halogenated nucleoside triphosphates such as 5-bromouridine-5′-triphosphate (BrUTP) which are detected by specific antibodies, a wide range of possible downstream applications emerged (Gratzner, 1982; Dundr and Raska, 1993; Jensen et al., 1993; Wansink et al., 1993; Chang et al., 2000). However, BrUTP is not absorbed well by living cells, and thus it has to be applied on isolated nuclei in so-called run-on assays (Thompson et al., 1997; Dhoondia et al., 2017), or introduced into cells via transfection, injection, or electroporation (Waksmundzka and Debey, 2001). Molecules such as 5-bromouridine (BrU), 5-iodouridine (IU), or 5-fluorouridine (FU), on the other hand, are efficiently taken up by living organisms. Direct incubation of fish in FU containing sea water or its injection into rats enabled tracking of RNA transcription *in vivo* (Casafont et al., 2006; So et al., 2010). Similar approaches also led to the development of genome-wide analyses of nascent RNA, isolated via chromatin immunoprecipitation using anti-BrU antibody. This method is called the BrU immunoprecipitation chase (BRIC) assay and involves deep sequencing of the obtained RNA moieties (Tani and Akimitsu, 2012; Imamachi et al., 2014).

In *A. thaliana*, the application of BrU has not been reported so far and the above-mentioned applications remain to be explored further. One of the few studies using BrU in plants by the run-on method was implemented in *Brassica napus* to describe nuclear transcription (Straatman et al., 1996). BrU combined with electron microscopy helped to uncover transcription in particular domains inside the nucleolus of garden peas (Thompson et al., 1997) and recently it has been successfully applied in tobacco (Singh et al., 2017).

Because BrU, FU, or IU are detected indirectly by immunofluorescence, the novel derivative 5-ethynyl uridine (EU), which can be revealed by a click iT reaction, brought a great improvement by reducing the number of steps in the detection procedure (Dimitrova, 2011). EU was shown to be incorporated efficiently into all kinds of RNA, and HPLC revealed that every 35th nucleotide is substituted by EU (Jao and Salic, 2008). Also, relatively short pulses (∼10 min) were sufficient to obtain visible signal in cultured cells.

The click iT reaction is a selective alkyne-azide cycloaddition where the ethynyl group of EU is covalently connected to azide-containing molecules under Cu (I) catalysis (Rostovtsev et al., 2002; Tornoe et al., 2002). Individual components of click iT reactions are small, which enables their use even in whole tissues including fixed whole root tips. Because the free copper (I) present in the click iT can affect other fluorescent molecules and precludes protocols where multiple labeling is needed (Kennedy et al., 2011; Dvorackova et al., 2018), picolyl azide in combination with copper chelates without any side effects were developed, as discussed previously (Kuang et al., 2010; Uttamapinant et al., 2012).

The click iT chemistry is nowadays widely used to label replication sites by ethynyl deoxy uridine (EdU). EdU was already successfully applied in *Arabidopsis*, first to visualize the DNA replication phase in cultured cells (Kotogany et al., 2010; Mickelson-Young et al., 2016), and later to track S phase progression in root meristems (Hayashi et al., 2013; Yokoyama et al., 2016; Dvorackova et al., 2018) or to detect proliferation capacity (Kazda et al., 2016). It was also demonstrated that EdU persists in plant material during growth and that it could be used to track cell fate (Watson et al., 2016).

As mentioned above, the use of EU remains to be explored in plants, and here we suggest to implement EU as an *in situ* marker of plant nucleoli. The nucleolus delimits the nuclear territory of transcriptionally active and mostly de-condensed ribosomal genes, corresponding to approximately 1 Mb in *A. thaliana* (Pruitt and Meyerowitz, 1986; Beven et al., 1995; Pontvianne et al., 2013). More traditional methods to label the plant nucleolus implement tagging of specific nucleolar proteins by fluorescent tags or raising antibodies against nucleolar proteins (e.g., Pendle et al., 2005; Pontvianne et al., 2007; Chandrasekhara et al., 2016). In addition, due to its relatively low DNA density the nucleolus does not stain well with 4′,6-diamidino-2-phenylindole dihydrochloride (DAPI) and it appears as a black hole inside the nucleus. Thus, the visualization of the nucleolus using EU is a relatively easy and fast approach, advantageous over many more demanding protocols and time-consuming protocols.

## Materials and Methods

WT-Col0 plants and plants expressing fibrillarin-YFP (kindly provided by F. Pontvianne, CNRS, Perpignan, France) were used. Plants were grown on aaa Murashige–Skoog (MS; Duchefa 0255) plates with 1% plant agar (Duchefa) and 1% sucrose. The growth conditions were: 8 h/16 h light/dark, 22°C, and light intensity 100 μmol m^-2^ s^-1^.

### EU Labeling

Two types of EU were used in this study, product CLK-N002-10 (Jena Bioscience, 200 mM in sterile water) and E-10345 (Life Technologies, 100 mM in DMSO). Four days old *A. thaliana* seedlings were transferred into 12-well plates (Greiner Bio-One). Each well contained 2 ml of the liquid growth medium (aaa MS). When CLK-N002-10 product was used, 20 μl of DMSO was added to the media to keep the same conditions as for the E-10345 product. Although DMSO is not required for efficient EU labeling, when the product E-10345 (diluted in DMSO) is used, seedlings are always exposed to 1% DMSO. Thus, when developmental studies or long EU incubation are performed, DMSO should be kept as low as 0.1% to avoid its potential side effects on the root growth (Shibasaki et al., 2009; Zhang et al., 2016). In such cases, the use of CLK-N002-10 product (diluted in water) is recommended. Alternatively, product E-10345 can be prepared as 0.5 M stock solution to decrease the DMSO content in the media.

Seedlings were labeled by adding 10 μM, 50 μM, 500 μM, or 1 mM EU into the liquid growth medium and incubated for the required time. The incubation was performed avoiding direct light.

### Fixation

5-Ethynyl uridine-labeled seedlings were fixed in freshly made 4% formaldehyde/1× PBS/0.5% Triton X-100 solution for 20 min, followed by 4% formaldehyde/1× PBS/1% Triton X-100 for an additional 25 min. The first 2 min of fixation was performed under vacuum (0.7 bar) in a plastic desiccator (Kartell). The 1× PBS buffer contained 137 mM NaCl, 2.7 mM KCl, 10 mM Na_2_HPO_4_, and 1.8 mM KH_2_PO_4_, pH 7.4. Formaldehyde solution (8%) was made by dissolving 0.8 g of paraformaldehyde (Sigma P6148) in 10 ml of distilled water containing 100 μl of 1 M NaOH and heated up to 60°C in the exhaust hood; the pH was then adjusted to 8.0 and the solution was filtered using Whatman filter paper. After fixation, seedlings were washed for 1 × 10 min in 1× PBS, 1 × 10 min in 1× PBS/135 mM glycine, and 10 min in 1× PBS, and proceeded directly to the click-iT reaction.

### Click iT Reaction

5-Ethynyl uridine-labeled and fixed seedlings were incubated with 500 μl–1 ml of click iT mixture containing 1× PBS, 4 mM CuSO_4_, 5 μM AF488 azide (Thermo Fisher Scientific, A10266), and 40 mM sodium ascorbate (Applichem A5048.0100, freshly prepared as a 400 mM solution and added into the click iT mixture at the required amount). The reaction was incubated for 1 h at room temperature in the dark and followed by two 5 min washes in 1× PBS. Alternatively, an Alexa Fluor 488 picolyl azide 488 toolkit (Thermo Fisher Scientific C10641) was used instead of the Alexa Fluor 488 azide protocol. This toolkit was developed to avoid quenching of fluorescent molecules caused by free copper present in the click iT reaction. It employs picolyl azide instead of azide and a protected copper solution. We efficiently used the picolyl azide provided in the C10641 kit as well as picolyl azide sulfo Cy5 (Jena Bioscience, CLK-1177). The reaction mixture was prepared according to the manufacturer’s protocol, using a copper:protected copper ratio of 1:1.

### DAPI Staining

4’,6-Diamidino-2-phenylindole dihydrochloride (DAPI, 1 mg ml^-1^; Serva) was added to the seedlings after performing the click iT reaction to a final concentration of 1 μg ml^-1^and incubated overnight in the refrigerator in the dark. The excess of DAPI was removed by two washes in 1× PBS. Shorter incubation with DAPI is recommended when the overnight incubation produces a high background noise.

### Nuclei Preparation

Overnight EU-labeled root tips were excised and fixed in freshly made ice-cold ethanol:acetic acid (3:1) fixative for 24 h. The fixative was exchanged once during this time. Roots were then washed 1 × 5 min in distilled water, 2 × 5 min in 10 mM citrate buffer (4 mM citric acid and 6 mM sodium citrate, pH 4.5), and digested by a mixture of cellulase (Onozuka R10, Serva 16419.03), pectolyase (Duchefa, P8004.0001), and cytohelicase (Sigma, C8274), 0.3% (w/v) each in 10 mM citrate buffer, for 25 min at 37°C. Digested root tips were washed once in citrate buffer and transferred to slides. After complete removal of the citrate buffer, root tips were squashed in a drop of 50% acetic acid. Cover slips were removed in liquid nitrogen, and slides were re-fixed in fresh ethanol:acetic acid fixative and air dried. The click iT reaction to detect EU by fluorescence was performed as described above, 200 μl of click iT mixture was applied on each slide. Slides were then washed 3 × 5 min in 1× PBS and stained with DAPI in Vectashield (1 μg ml^-1^, Vector Laboratories, H100).

### Actinomycin D Treatment

To inhibit transcription, Actinomycin D (ActD, Sigma, A1410, 1 mg ml^-1^ in DMSO) was added to the aaa MS/0.5% sucrose liquid medium in 6-well plates (Greiner Bio-One) to a final concentration of 25 μg ml^-1^. Four days old seedlings were incubated for 2 h with ActD, then for 2 h with 1 mM EU, and processed as stated above.

### Microscopy

Root tips were transferred onto slides with a drop of water, covered with coverslips, and imaged on a Zeiss LSM780 confocal microscope using a 40× C-Apochromat/1.20 W objective and *Z*-stacks of 1.0–1.4 μm step size, pinhole 66–68 μm. Alternatively, a Zeiss Axioimager Z1 with filters corresponding to DAPI and AF488 excitation and emission spectra (AHF Analysentechnik^1^) was used. Image processing was done in ImageJ^2^.

## Results and Discussion

### EU Labeling of Nucleolar Processes

#### Visualization of Nucleolar Transcription

The majority of RNA transcripts in the plant nucleus correspond to the rRNA genes. RNA polymerase I, the enzyme responsible for rRNA transcription inside the nucleolus, can be efficiently blocked by ActD leading to the re-distribution of nucleolar proteins and nucleolar fragmentation (Yung et al., 1990; Chen and Jiang, 2004). Efforts to detect rRNA synthesis by qPCR after ActD treatment are biased, likely due to pleiotropic effects of ActD on other RNAs including transcripts of the reference genes, as discussed (e.g., Turner et al., 2012). Here, we present an assay to detect rRNA transcription *in situ* using 5-EU and test the protocol on ActD-treated seedlings.

#### Overnight EU Labeling

**FIGURE 1:**
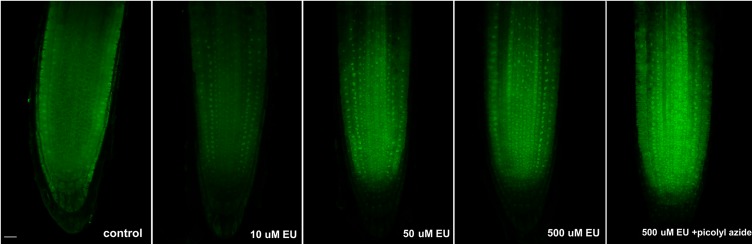
Long EU pulses. *Arabidopsis* 4 days old seedlings were incubated overnight avoiding direct light with an increasing concentration of EU (Jena Bioscience) and EU-containing RNA was detected by the click iT reaction. The last image in the row shows the result of a modified detection protocol using AF488 picolyl azide instead of AF488 azide. Confocal sections in the middle part of the root are shown. Bar = 10 μm.

Initially, EU was applied on 4 days old seedlings at different concentrations (10, 50, and 500 μM), incubated overnight, and detected by the click iT reaction (**Figure 1**). The fixation step in the protocol included incubation of seedlings with higher concentration of Triton X-100 (compared to standard protocols) to facilitate the penetration of the click iT components into the nucleolus. All labeling pulses showed a similar labeling pattern, and a small round area inside the nucleus was observed in each cell, as expected for a nucleolar signal (**Figure 1**). A better signal-to-noise ratio was achieved when lower EU concentrations were used, probably indicating that an excess of EU contributes to the background noise signal or that the signal becomes re-distributed. The presence of cytoplasmic signal was also observed in other tested species after long incubation likely reflecting the RNA dynamics in the cell (Jao and Salic, 2008).

We next tested whether the modified version of the click iT reaction using picolyl azide and protected copper (instead of azide and reactive copper species) that is required when quenching has to be inhibited (e.g., in double labeling protocols including fluorescently tagged proteins or flow cytometry) was similarly efficient in EU detection. As shown in **Figure 1**, the modified click iT reaction produced satisfactory signal intensity similar to the standard click iT detection method. This shows that in addition to visualization of the nucleolus, the method could be efficiently used, e.g., in flow cytometry or for double labeling protocols. Since the size of nucleoli differs in different cell types, the protocol could be further exploited to measure, for example, the size of the nucleoli. Also, in combination with fluorescence-activated cell sorting, rDNA transcription can be further evaluated at the single cell level.

#### Short EU Pulses

**FIGURE 2:**
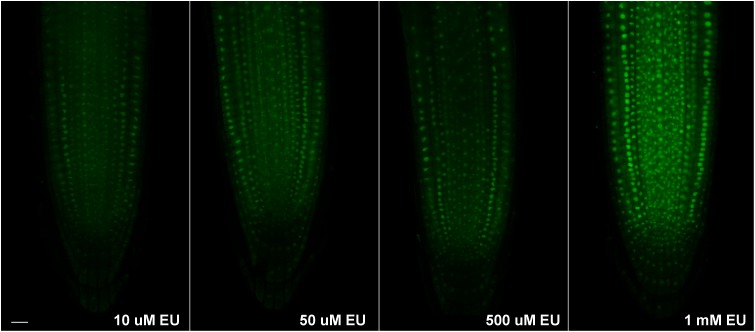
Short EU pulses. *Arabidopsis* 4 days old seedlings were incubated avoiding direct light for 2 h with an increasing concentration of EU (Jena Bioscience) and EU-containing RNA was detected by the click iT reaction. Confocal sections in the middle part of the root are shown. Bar = 10 μm.

To allow for detection of ongoing transcription, shorter EU pulses were necessary. Thus, the EU labeling was repeated again with three different concentrations of EU (10, 50, and 500 μM) and the labeling time shortened to 2 h. While 10 μM EU produced a rather weak signal, 50 and 500 μM EU were brighter (**Figure 2**). The signal-to-noise ratio, however, was not satisfactory and needed improvement. Finally, the optimal concentration for short EU pulses was set to 1 mM (**Figure 2**) which provided the expected result. The EU signal appeared not only in the root tip, but also in some leaf cells as shown in Supplementary Figure 1. Since the signal was not seen in hypocotyls, it is likely that leaves can absorb EU via stomata.

**FIGURE 3:**
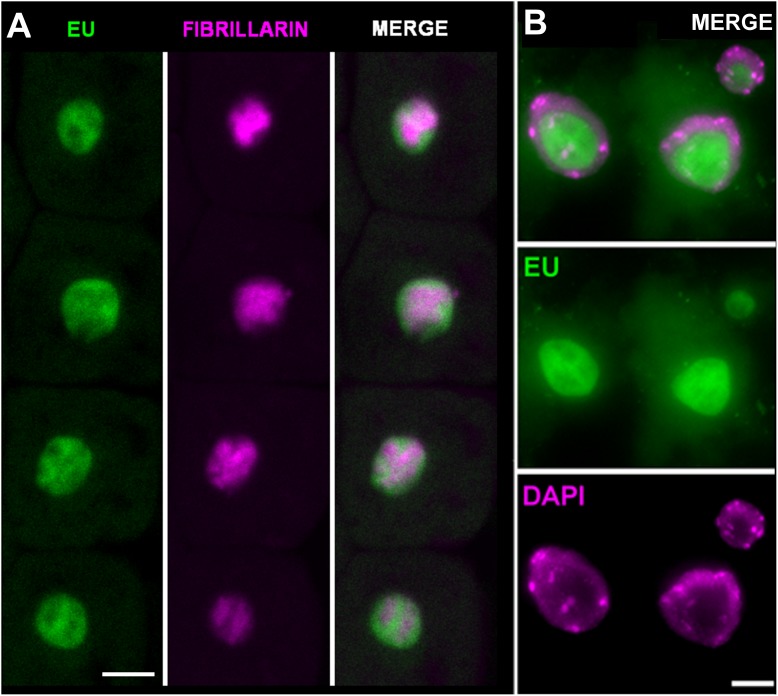
Ethynyl uridine-containing RNA accumulates in the nucleolus. **(A)** *Arabidopsis* seedlings expressing fibrillarin-YFP (magenta) were labeled with 1 mM EU (Invitrogen, green) for 2 h and EU-containing RNA was detected by the click iT reaction. Selected sections from confocal *Z*-stacks are shown. Bar = 5 μm. **(B)** Cytological spread of EU (green) and DAPI (magenta) labeled nuclei from root tip incubated avoiding direct light with EU overnight. The detailed nucleolar structure is detectable. Bar = 5 μm.

To confirm in more detail where the observed EU signal accumulates, two additional experiments were performed. First, fibrillarin-YFP expressing plants were EU labeled and signal overlap between the fibrillarin and EU was assessed (**Figure 3A**). Second, double EU/DAPI was applied along with EU labeling (**Figure 3B**). The best DAPI signal was achieved by overnight incubation of fixed seedlings with a low DAPI concentration (1 μg ml^-1^) followed by two washes with 1× PBS.

Both experiments show that the majority of the detectable signal occurs in the nucleolus, and the DAPI staining confirmed that signal in the nucleoplasm cannot be detected. The nucleolar signal does not entirely overlap with fibrillarin. In fact, it is largely accumulated in areas with a lower fibrillarin density and expands outside the area delimited by the fibrillarin. This EU distribution seems to reflect compartmentalization of the processes in the nucleolus. Pre-rRNA is mostly transcribed at the periphery of the fibrillar centers, while fibrillarin occurs in the dense fibrillar component (see e.g., Jordan, 1984; Ochs et al., 1985; Beven et al., 1995; de Carcer and Medina, 1999; Raska et al., 2006; Montanaro et al., 2011).

It is interesting that a similar labeling pattern is achieved using short or long EU pulses and that the EU signal is detectable exclusively inside the nucleolus and in its vicinity. These results are contrary to the EU pattern observed in cell cultures, where nuclear signal is detected along with strong nucleolar labeling (Jao and Salic, 2008). Also, the same study reported that the rRNAs are labeled with a lower efficiency, while labeling of poly(A)-containing mRNAs was more profound. In our *in situ* experiments, it seems that the rRNA fraction is the only labeled RNA. This might suggest a relatively high turnover of labeled RNA in *Arabidopsis* cells, or a sensitivity issue in the protocol. It may be necessary to amplify fluorescent signal by biotin–streptavidin system or antibodies to reveal remaining RNA transcripts. Also, during the fixation, higher amounts of Triton X-100 were used, which could possibly cause the re-distribution of nuclear RNAs into the cytoplasm. It is possible that signal would be more stabilized if the detection is performed on isolated nuclei instead of the whole root, requiring stronger permeabilization step in the protocol. We confirmed that RNA turnover was very fast by pulse-chase experiment. When we incubated seedlings for 2 h in EU followed by 3 or 6 h incubation in EU-free aaa MS medium, no signal was detected (Supplementary Figure 2). Although decreased stability of EU-containing RNA in plant tissue has not been reported, it could not be completely neglected. We observed, for example, that when EU-labeled material is stored, after some time the signal diminishes, but when similar material is stored after EdU labeling, this phenomenon does not occur. We also tested whether light could affect the EU stability. However, incubation of seedlings in dark or light does not seem to have any strong effect on EU labeling (Supplementary Figure 3).

#### Actinomycin D Blocks Nucleolar Transcription

**FIGURE 4:**
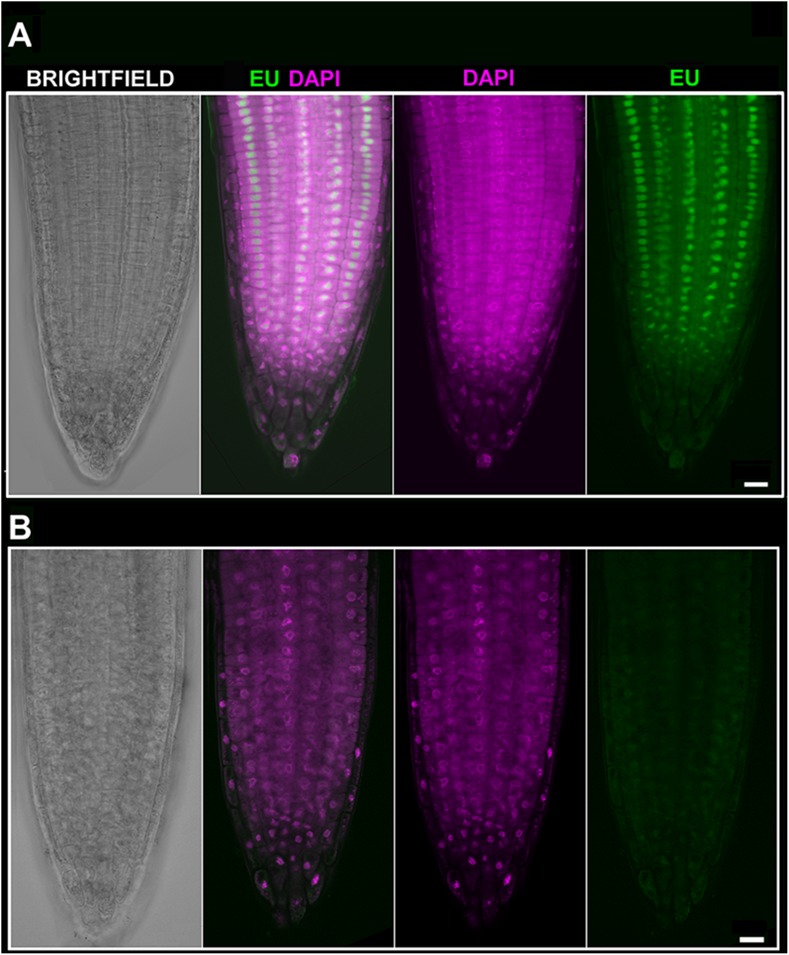
EU signal diminishes after Actinomycin D (ActD) treatment. **(A)** *Arabidopsis* 4 days old seedlings were incubated without pre-treatment for 2 h with 1 mM EU (Invitrogen, green) avoiding direct light, detected by click iT reaction and stained with DAPI (magenta). Confocal sections in the middle part of the root are shown. Bar = 10 μm. **(B)** *Arabidopsis* 4 days old seedlings were treated for 2 h with ActD prior to EU labeling. DAPI (magenta), EU (green). Selected sections from confocal *Z*-stacks are shown. Bar = 10 μm.

**FIGURE 5:**
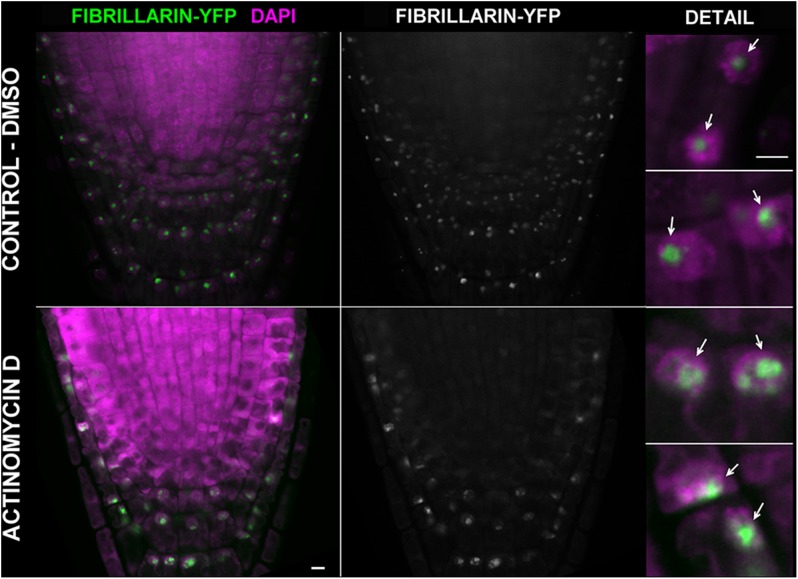
Actinomycin D causes re-distribution of fibrillarin. *Arabidopsis* 4 days old seedlings expressing fibrillarin-YFP (green) were treated with 25 μg ml^-1^ of ActD for 2 h (the bottom panel). In the upper panel, a control incubated in the solvent (2.5% DMSO) is shown. Roots were fixed, stained with DAPI (magenta), and imaged on a confocal microscope. Selected sections from confocal *Z*-stacks are shown. Arrows point to the nucleoli. Bar = 5 μm.

We next asked whether inhibition of transcription could be monitored by using EU. The effect of ActD on RNA pol I which further changes the nucleolar structure has been long known (Unuma et al., 1972; Kramer, 1980). Thus, we treated *Arabidopsis* seedlings with ActD prior to the EU incubation. When EU was added after 2 h of ActD treatment, no RNA transcripts were detected indicating that ActD acted in the expected manner (**Figure 4**). To further characterize the effect of ActD on plant nucleoli, nucleolar integrity was monitored by fibrillarin-YFP after ActD treatment (**Figure 5**). After 2 h of ActD, fibrillarin started to re-localize from the nucleolus, confirming the sensitivity of the nucleolus to this drug as well as proving that the EU signal corresponds to the nascent RNA transcripts.

As we demonstrate here, labeling of the nucleolus by 5-EU represents a reliable protocol applicable to monitor nucleolar transcription directly in the root tip. The protocol can be used to track potential rRNA transcription inhibitors or rRNA metabolism under various stress conditions. Due to the elevated protein density inside the nucleolus the procedure requires relatively high detergent concentrations. In addition, high EU concentrations are required when shorter EU pulses are used. Thus, possible side effects need to be considered in each experimental set-up.

### Conclusion

Developments in microscopic approaches and their combinations with tissue- and cell-type-specific labeling and nuclei sorting allow for description of previously unknown details of key cellular processes *in situ* or *in vivo* at a high spatiotemporal resolution. This new knowledge is obtained at the cost of three factors: the increasing complexity of experiments, the high cost of instrumentation, and the need for careful optimization of methods for a given purpose and model system. While the first two factors can be efficiently managed in current well-established research centers, optimization remains challenging and the most time-consuming part of these experiments. Therefore, we describe here the optimized approach to visualize transcription in nucleoli of *A. thaliana in situ* to share this experience with the plant science community.

### Author Contributions

MD designed the experiments and performed the optimization of the protocol and all experiments. JF and MD contributed to the concept of the project and data interpretation, prepared the manuscript, and co-supervised the project.

## Conflict of Interest Statement

The authors declare that the research was conducted in the absence of any commercial or financial relationships that could be construed as a potential conflict of interest.

## Footnotes

1 http://www.ahf.de/

2 http://imagej.nih.gov/ij/
