# Supplementary material for: Visualization of the Nucleolus Using Ethynyl Uridine
Source: Front Plant Sci. 2018 Feb 16;9:177. doi: 10.3389/fpls.2018.00177 (PMC5820300; doi:10.3389/fpls.2018.00177)
Supplement: Supplementary file 1 [file Image_1.pdf]

Supplementary Material

Visualization of the nucleolus using ethynyl uridine

Martina Dvořáčková and Jiří Fajkus

*** Correspondence:** jiri.fajkus@ceitec.muni.cz, dvorackova.martina@gmail.com

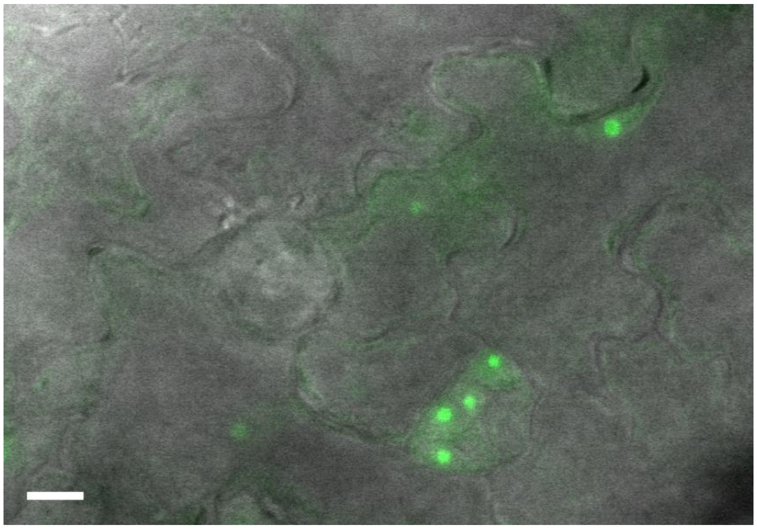

Supplementary Figure 1. EU signal can be detected in the leaf cells.

Arabidopsis 4 days old seedlings were incubated for 2 hr with 1 mM EU, fixed and EU-containing RNA (green) was detected by the click iT reaction, bar= 10 µm

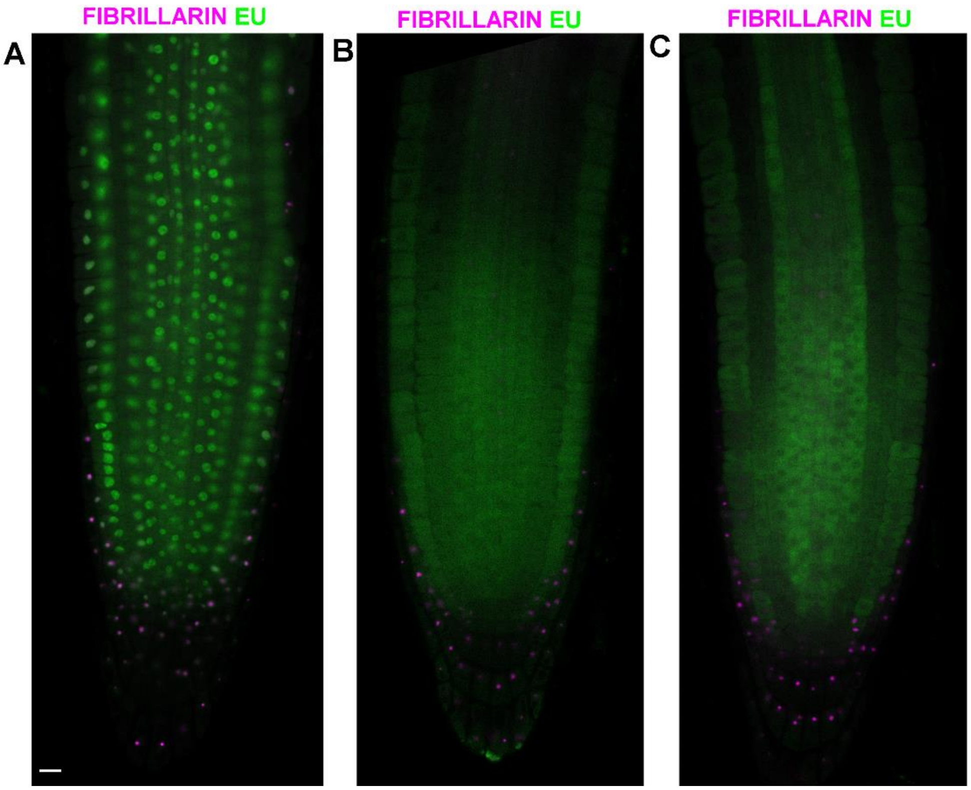

Supplementary Figure 2. EU-labelled RNA shows fast turnover.

- **(A)** *Arabidopsis* 4 days old seedlings expressing fibrillarin-YFP (magenta) were incubated for 2 h with 1 mM EU, fixed and EU-containing RNA (green) was detected by the click iT reaction.
- **(B)** *Arabidopsis* 4 days old seedlings expressing fibrillarin-YFP (magenta) were incubated for 2 h with 1 mM EU followed by incubation in EU-free medium for 3 h. EU-labelled seedlings were fixed and EU-containing RNA (green) was detected by the click iT reaction.
- **(C)** *Arabidopsis* 4 days old seedlings expressing fibrillarin-YFP (magenta) were incubated for 2 h with 1 mM EU followed by incubation in EU-free medium for 6 h. EU-labelled seedlings were fixed and EU-containing RNA (green) was detected by the click iT reaction.

Bar=10 µm

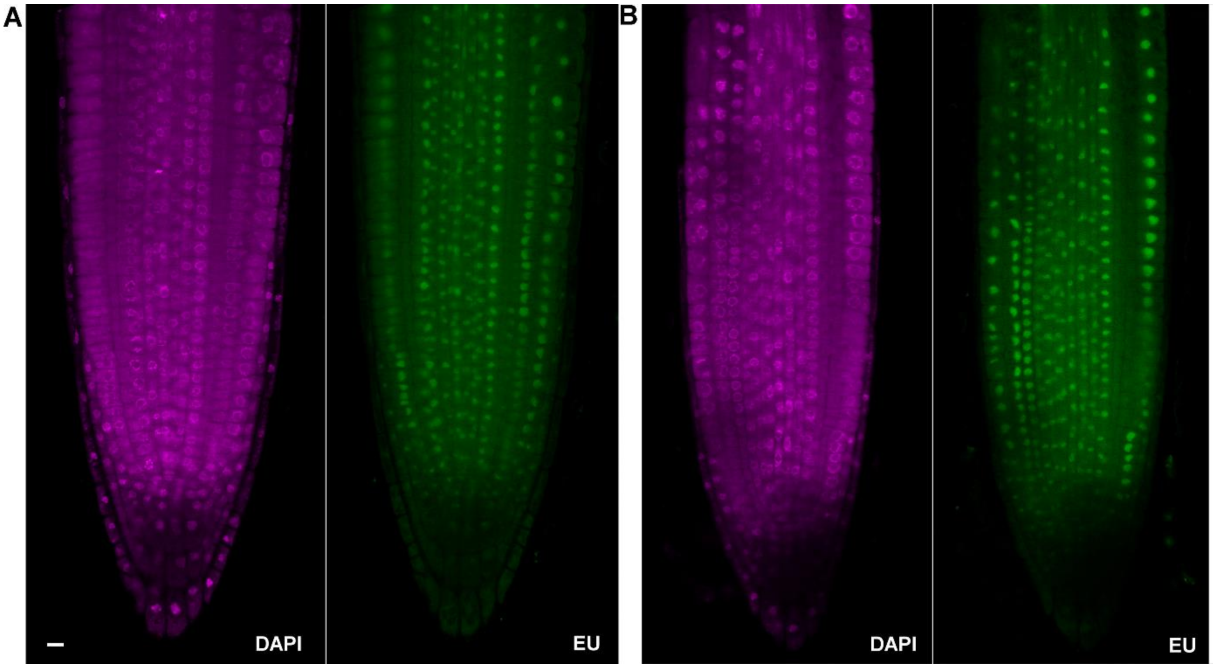

Supplementary Figure 3. Negligible effect of incubation in the dark or the light.

Arabidopsis 4 days old seedlings were incubated for 2 h with 1 mM EU (green) in the dark **(A)** or in the light **(B),** fixed and EU-containing RNA was detected by the click iT reaction, bar= 10 μm
